# Hypoxia Pretreatment of Bone Marrow Mesenchymal Stem Cells Facilitates Angiogenesis by Improving the Function of Endothelial Cells in Diabetic Rats with Lower Ischemia

**DOI:** 10.1371/journal.pone.0126715

**Published:** 2015-05-21

**Authors:** Jiejie Liu, Haojie Hao, Lei Xia, Dongdong Ti, Hong Huang, Liang Dong, Chuan Tong, Qian Hou, Yali Zhao, Huiling Liu, Xiaobing Fu, Weidong Han

**Affiliations:** 1 Institute of Basic Medicine Science, College of Life Science, Chinese PLA General Hospital, Beijing, China; 2 Central laboratory, Hainan branch of Chinese PLA General Hospital, Sanya, China; 3 Department of Medical Administration, Chinese PLA General Hospital, Beijing, China; University of Torino, ITALY

## Abstract

Endothelial dysfunction induced by unordered metabolism results in vascular reconstruction challenges in diabetic lower limb ischemia (DLLI). Mesenchymal stem cells (MSCs) are multipotent secretory cells that are suitable for clinical DLLI treatment, but their use has been hampered by poor survival after injection. Hypoxia can significantly enhance the capacity of MSCs to secrete angiogenic factors. We investigated transient hypoxia pretreatment of MSCs to facilitate revascularization in DLLI. Rat bone marrow MSCs (BM-MSCs) were cultured at different oxygen concentrations for varying time periods. The results indicated that transient pretreatment (5% O_2_, 48 h) not only increased the expression of VEGF-1α, ANG, HIF-1α and MMP-9 in BM-MSCs as assessed by real-time RT-PCR, but also increased the expression of Bcl-2 as determined by western blotting. The transplantation of pretreated BM-MSCs into rats with DLLI demonstrated accelerated vascular reconstruction when assayed by angiography and immunohistochemistry. CM-Dil-labeled tracer experiments indicated that the survival of BM-MSCs was significantly improved, with approximately 5% of the injected cells remaining alive at 14 days. The expression levels of VEGF-1α, MMP-9 and VEGF-R were significantly increased, and the expression of pAKT was up-regulated in ischemic muscle. Double immunofluorescence studies confirmed that the pretreated BM-MSCs promoted the proliferation and inhibited the apoptosis of endothelial cells. In vitro, pretreated BM-MSCs increased the migratory and tube forming capacity of endothelial cells (ECs). Hypoxia pretreatment of BM-MSCs significantly improved angiogenesis in response to tissue ischemia by ameliorating endothelial cell dysfunction and is a promising therapeutic treatment for DLLI.

## Introduction

Lower limb ischemia, a common complication in Type 2 diabetes (T2D), afflicts approximately 15% of the diabetic patient population worldwide [1, 2]. The dysregulation of vascular remodeling and vascular growth is induced by hyperglycemia and results in damage to endothelial cells (ECs) [3, 4]. The dysfunction of ECs generates decreased responsiveness to ischemic/hypoxic stimuli, impaired or abnormal neovascularization, and a lack of endothelial regeneration and is associated with peripheral microvascular complications in diabetes [5]. Thus, a key strategy for restoring blood flow is the repair of dysfunctional ECs.

Previous reports have demonstrated the positive effects of ECs in vascular network reconstruction in terms of angiogenesis for lower limb ischemic lesions [6]. The complicated pathophysiological changes that occur as a result of diabetes could exacerbate the functional damage of ECs, contributing to impaired re-endothelialization and vascular recovery. Some strategies attempt to recover the mobilization, adhesion, tube formation and proliferation of ECs by modifying relevant molecular pathways, including the eNOS, CXCR-4 and hemeoxygenase-1 (HO-1) pathways [7–9]. Other proangiogenesis factors such as VEGF, HGF, and proangiogenesis protein MMP-9 repair endothelial dysfunction by improving the ability of ECs to migrate and proliferate [10–12]. Enhanced angiogenesis after VEGF treatment was accompanied by the increased expression of the adhesion protein N-cadherin, which mediates endothelial-pericytic interactions, in ischemic brain microvessels [13]. However, continuous hyperglycemia causes defective VEGF signaling, including the inactivation of the VEGF receptor FLK-1 [14], which affects endothelial growth and migration as well as monocytes and ECs recruitment and released from bone marrow in DLLI. Due to defective VEGF signaling that results in the low bioavailability of VEGF, the effect of providing VEGF to promote angiogenesis should further explored.

MSCs are able to function as paracrine or autocrine signals that induce the release of proangiogenic cytokines, resulting in the stimulation of resident ECs to migrate, differentiate, and proliferate *in situ*, and are thought to influence neovascularization. *In vitro*, MSCs secrete VEGF and other angiogenic factors that can promote endothelial cell migration, proliferation and tube formation [15]. *In vivo*, the mechanisms underlying MSCs improvement of cardiac functions may involve neovascularization that induced by the parasecretion of growth factors in myocardial infarcted rats with occlusion of the left anterior descending artery [16]. Furthermore, to increase the therapeutic potential of MSCs, alternative methods have been investigated to enhance their ability to secrete proangiogenic factors. For instance, the transplantation of VEGF-1α gene-transfected MSCs improved the treatment of myocardial perfusion and the restoration of heart function in ischemic heart models of inbred rats [17, 18]. To avoid the risk of genetic modification, overexpressed the angiogenic factors on MSCs to promote angiogenesis after DLLI treatment.

The O_2_ concentration of MSCs in the bone marrow is 2%-7% under physiologic conditions [19, 20]. *In vitro*, MSCs are cultured at an ambient O_2_ concentration (21% O_2_), which is unsuitable for secretion activity. Once the cells that expanded *in vitro* were transplanted and localized to the ischemic tissue, the MSCs encountered severely hypoxic conditions, ranging from 0.4% to 2.3% O_2_, which often resulted in apoptosis [21]. Previous studies have demonstrated that hypoxia-induced apoptosis can be circumvented by preconditioning cells in less severe hypoxic conditions for a period of time before exposing them to severe ischemia at the site of injury in other cell types. MSCs exposed to a hypoxic cultural environment at 0.5% O_2_ after 24 h and 48 h were in a state of prosurvival and adaptation [22]. Research exploring the mechanism shows that MSCs cultured under hypoxic conditions and transplanted to a subcutaneous polyvinyl alcohol sponge model exhibited increased paracrine production of VEGF and ANG [23]. The reports suggested that, in addition to maintaining their viability when cultured in physiologic niche conditions, MSCs also increased their secretion after an initial lag phase [24, 25]. Therefore, an exploration of the effects of an appropriate physiological environment on the ability of MSCs to secrete proangiogenic factors and maintain quiescence when transplanted into ischemic tissue is worthwhile.

In our study, we identified that a suitable physiologic niche treatment of 5% O_2_ for 48 h for BM-MSCs significantly increased the secretion of proangiogenesis factors and enhanced angiogenesis in ischemic tissue by ameliorating endothelial dysfunction for DLLI treatment.

## Materials and Methods

### Isolation and culture of BM-MSCs

Sprague–Dawley (SD) rats were purchased from the Chinese PLA General Hospital and were housed under specific pathogen-free conditions in the animal center of Chinese PLA General Hospital. All animal experiments were carried out in accordance with the National Institutes of Health Guide for Care and Use of Laboratory Animals and were approved by the Animal Care and Use Committee at the Chinese PLA General Hospital. MSCs were generated from the bone marrow of adult male SD rats, aged 4–6 weeks and weighing 120–150 g, according to a previously published protocol [26]. Briefly, the bone marrow aspirate was obtained by flushing the rat femurs and tibias with fetal bovine serum (FBS)-free low glucose (LG)-DMEM medium (Invitrogen, Carlsbad, USA). Then, the aspirate samples were centrifuged and re-suspended in LG-DMEM medium with 10% FBS and incubated at 37°C in a humidified chamber with 5% CO_2_. The culture media was completely replaced every 3 days, and the non-adherent cells were discarded. The MSCs were recognized by their ability to proliferate in culture with an attached well-spread morphology. Once the cells were more than 80% confluent, the adherent cells were detached and re-plated at a 1:3 dilution in culture flasks.

### Cell apoptosis by flow cytometry

For hypoxia treatment, the cells were subcultured at a 1:4 dilution and cultured for 3 days until confluent. Fresh complete medium was added prior hypoxia treatment, which was performed in a well characterized, finely controlled pro-C-O—chamber (Thermo) for 24 h. The oxygen concentration in the chamber was maintained at 2%, 5% or 7% with a residual gas mixture composed of 5% CO_2_ and balanced nitrogen. After hypoxia pre-conditioning, the MSCs were collected, washed with PBS, resuspended in PBS at 1×10^6^/mL, and stained with Annexin V and propidium iodide solution (PI, BD Biosciences Pharmingen, San Diego, USA) for 15 min in the dark. Apoptotic cells were then analyzed by flow cytometry (BD Biosciences Pharmingen, San Diego, USA).

### Real-time RT-PCR

Total RNA was extracted from MSCs using Trizol reagents (Invitrogen, Carlsbad, USA) according to manufacturer’s instructions. Reverse transcription was performed using a Takara RT-PCR Kit (Takara, Japan). Real-time PCR was performed using SYBR Premix Ex Taq (Takara, Japan) and a 7500 Real Time PCR System (Applied Biosystems, Foster City, USA) according to standard protocols. To control for equal input levels, β-actin mRNA was determined and the data were expressed as ratios relative to the x-actin mRNA levels. The sequences of the primers used are listed in Table 1.

**Table 1:** List of primers used for real-time RT-PCR.

Gene name	Forward(5’- 3’)	Reverse(5’- 3’)
PDGF	GTCCAGGTGAGGTTAGAGG	CACGGAGGAGAACAAAGAC
HIF-1α-F	AAGTCTAGGGATGCAGCAC	CAAGATCACCAGCATCTAG
VEGF-1a	CAGCTATTGCCGTCCAATTGA	CCAGGGCTTCATCATTGCA
Mmp-9	TGACATCTATGCAATGGGCTTAGTAT	CTTGGTGGATTVVGCCAAT
HGF	GAGGAGAAACGCAAACAG	ACGACCAGGAACAATGAC
β -actin	GAG ACC TTC AAC ACC CCA GCC	AATGTCACGCACGATTTCCC

### Rat diabetic and ischemic hind limb model

To induce moderate diabetes, SD rats (8-weeks old, n = 25) were injected intraperitoneally with 65 mg/kg of streptozotocin (STZ, Sigma, St. Louis, MO, USA) in 0.9% sterile saline daily for 3 days. Blood glucose levels were monitored at 0, 3, 5, and 7 days after the initial injection. Rats with glucose levels less than 250 mg/dL after 3 days of STZ treatment were excluded from further studies. The diabetic rats then underwent unilateral hind limb ischemia 7 days after the initial STZ injection. Briefly, after intraperitoneal injection of 3% sodium pentobarbital anesthesia, a longitudinal incision was made along the left medial thigh to allow the isolation, ligation, and excision of the femoral artery from its origin directly above the inguinal ligament to its bifurcation at the origin of the popliteal and saphenous arteries.

### Local transplantation of BM-MSCs

BM-MSCs for transplantation were labeled with CM-Dil (Invitrogen, Carlsbad, USA) according to the manufacturer's instructions. The ischemic, diabetic animals were immediately randomly assigned into 3 groups and received intramuscular injections (100 μl in saline) of either 4×10^6^ of hypoxia-treated, Dil-labeled BM-MSCs; normoxic, Dil-labeled BM-MSCs; or a blank sample as a negative control. The injection points were evenly distributed among six injections in the ischemic muscle along the femoral artery. The skin incision was closed with 2–0 interrupted silk sutures (Ethicon), and all animals were closely monitored during the postoperative period.

### Angiography

The SD rats were anesthetized with sodium pentobarbital and fixed to the sample table. The abdominal cavity was opened, and a catheter was inserted downward from the abdominal aorta for the injection of the contrast medium. The animals were transferred onto an OEC9800 (GE, USA), and the X-ray was adjusted to the target area, followed by the injection of contrast agent through the indwelling catheter and instantaneous imaging.

### Laser Doppler perfusion imaging

Blood flow in DLLI was mapped with a high resolution pericam PSI Laser Doppler perfusion imager (Perimed Inc., NorthRoyalton). The parameter settings during measurement were as following: scanning area, 50 mm×50 mm; high resolution; distance between the scanner head and wound, 15 cm; temperature. The measurement of the image and perfusion value was carried out with LDISOFT software.

### Western blot analysis

Muscle tissues and co-cultured cells were lysed in protein lysis buffer (Sigma, St. Louis, MO, USA). The protein lysates were loaded onto 8%-15% sodium dodecyl sulfate polyacrylamide gels, and then, the separated proteins were transferred to polyvinylidene difluoride (PVDF) membranes, blocked, and incubated overnight at 4°C with primary antibodies. The membranes were washed and then incubated with a secondary antibody for 1 hour at room temperature using an orbital shaker. After washing, the bands were detected using an enhanced chemiluminescence reagent (Santa Cruz Biotechnology, Santa Cruz, USA).

### Histological analysis and immunohistochemistry

The rats were sacrificed by the intraperitoneal administration of an overdose of pentobarbital at the indicated time points, and the ischemic thigh muscles were carefully excised and post-fixed in 4% paraformaldehyde or paraffin. For morphometric analysis, the muscle fiber number and size were examined in sections stained with hematoxylin-eosin, with the averaging of the counts from 10 separate fields in 5 different areas of each specimen. For histological analysis of vascularization and cell surface markers, 5-mm paraffin sections were incubated for 16 h at 4°C in a 1:250 dilution of an anti-vWF antibody (Abcam., Cambridge, USA) with an antibody diluent (Invitrogen, Carlsbad, USA) and then for 60 min at RT with a 1:300 dilution of a biotinylated goat anti-rabbit IgG antibody (Invitrogen, Carlsbad, USA) with PBS, followed by development with DAB (Sigma-Aldrich, St. Louis, USA). Five fields from each tissue section were randomly selected, and the number of microvessels was counted. To analyze the mechanism through which the injected BM-MSCs promoted angiogenesis, serial frozen section were incubated overnight at 4°C with rabbit antibodies against either vWF (1:150 dilution in antibody diluent) or Ki67 (1:100 in antibody diluent; BD Biosciences Pharmingen, San Diego, USA); then, the sections were incubated with FITC-conjugated goat anti-rabbit IgG (1:200 dilution in PBS-NGS; Invitrogen, Carlsbad, USA) or FITC-conjugated goat anti-mouse IgG at 37°C for 1 h.

### TUNEL staining

Apoptotic cells were identified by terminal dUTP nick-end labeling (TUNEL) staining according to the manufacturer's protocol using a commercially available kit (Molecular Probes, USA). The sections were counterstained with Hoechst 33342 (Sigma-Aldrich, St. Louis, USA) to visualize all nuclei and were viewed under a fluorescence microscope (Olympus).

### Construction of siRNA-expressing vectors

VEGF-1α-specific target sequences were chosen according to online siRNA tools from Invitrogen using the VEGF-1α reference sequence. The target sequences for VEGF-1α are as follows: sense1 5'-CACCGGGATGACCTTGTAGTGAAGGCGAACCTTCACTACAAGGTCATCCC-3'; sense2 5'- CACCGCTGTGCCTTTCACAGTTTCTCGAAAGAAACTGTGAAAGGCACAGC-3'; 、antisense 5'-CACCGATACAGTAGTAGTAGGACCTGCGAACAGGTCCTACTACTACTGTA-3'. The siRNAs were chemically synthesized, and a lentiviral vector was constructed as described by the Invitrogen, Carlsbad, USA lentiviral vector protocol. The correct insertion of the specific siRNA was confirmed by sequencing (Invitrogen, Carlsbad, USA).

### Cell migration and tube formation assay

To examine the effect of BM-MSCs-conditioned media on the migration of HUVECs, an endothelial cell wound injury repair assay was used. HUVEC were purchased from China Center of Type Culture Collection (CCTCC) in Wuhan. The cells were cultured in RPMI 1640 medium complemented with 10% heat-inactivated fetal bovine serum, penicillin (100 IU/ml), streptomycin (100 mg/ml) and 2 mM L-glutamine in a humidified CO_2_ incubator with 5% CO_2_ at 37°C. HUVECs were grown in a 24-well dish, and wounds were inflicted by dragging a sterile pipette tip across the monolayer, creating a 350 mm cell-free path. The medium was then replaced with conditioned medium (CM) from BM-MSCs cultured in LG-DMEM under normoxia for 48 h (N-CM) or hypoxia for 48 h (H-CM) or from siVEGF-1α transfected BM-MSCs cultured under hypoxia (siVEGF-H-CM). After 12 h under normoxic conditions, the wound area/original wound area ratio was calculated using GraphPad Prism 5 Software. The vascular tube formation assay was performed by plating HUVECs in one well of a 96-well plate precoated with 40 mL of Matrigel (BD Biosciences Pharmingen, San Diego, USA); adding N-CM, H-CM, or siVEGF-H-CM; and then incubating the plates for 16 h at 37°C under normoxic conditions. Then, tube formation was examined under an inverted phase-contrast microscope, and the vessel length was analyzed.

### Statistical analysis

Statistics were performed using GraphPad Prism 5 Software. The data, collected from at least three independent experiments, are expressed as the means with standard errors. Statistical comparisons were made using one-way analysis of variance, and the results were considered to be significant statistically when *P* < 0.05.

## Results

### The characterization of rat BM-MSCs cultured under hypoxic conditions

**Fig 1:**
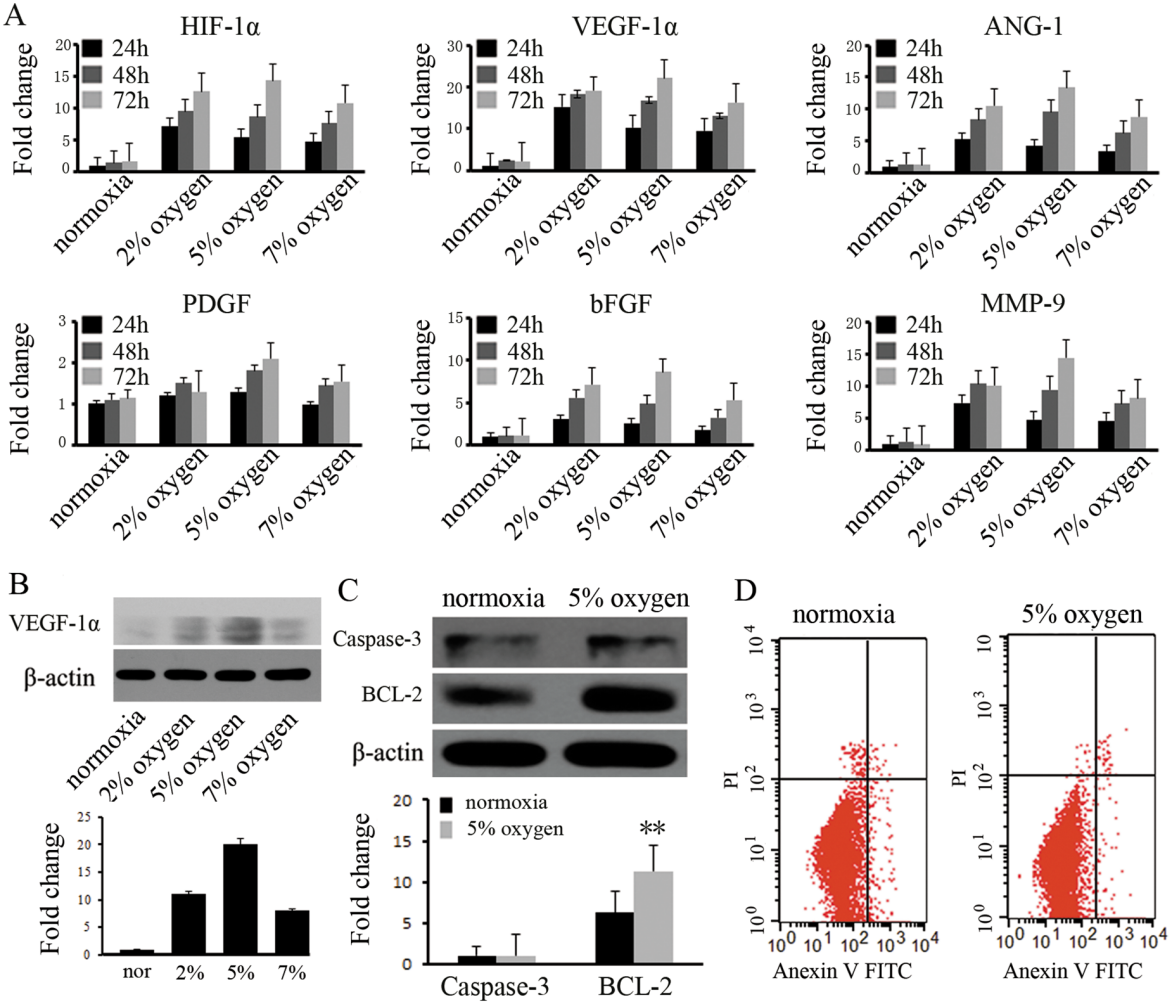
The characterization of rat BM-MSCs cultured under hypoxic conditions. (A) The expression of VEGF-1α, HIF-1α, ANG-1, bFGF, and MMP-9 were significantly increased in BM-MSCs receiving hypoxia pretreatment for 24 h compared to normoxic controls. At 48 h or 72 h, hypoxia pretreatment at 5% O_2_ continuously increased the expression of these genes. (B) The expression of the VEGF-1a protein was significantly increased in BM-MSCs after hypoxia pretreatment. (C) The expression of the anti-apoptosis protein Bcl-2 was enhanced by western blot. * indicates *P*<0.05 versus the normoxia group, and (D) the apoptosis of BM-MSCs was analyzed by flow cytometry for cells cultured with 5% O_2_. At 48 h, no changes were observed *in vitro*,.

To characterize rat BM-MSCs cultured under hypoxic conditions, BM-MSCs were isolated and cultured with different oxygen concentrations for different times. The results demonstrated that the expression of VEGF-1a, HIF-1a, HGF, bFGF, MMP-9, and PDGF was enhanced in BM-MSCs cultured under hypoxia (2%, 5%, or 7%), as determined by real time RT-PCR. However, we found that the expression of those genes was transient, with gene expression most markedly increased in BM-MSCs cultured in 5% O_2_ for 48 h. In particular, the expression of VEGF-1a and HIF was highly upregulated, compared to the other genes (Fig 1A). Western blotting showed that the expression of VEGF-1a was consistent with the gene expression results (Fig 1B). For this reason and because VEGF-1a and HIF play important roles in promoting angiogenesis, the BM-MSC pretreatment condition of 5% O_2_ for 48 h was chosen for this study. We further observed the transient effect of pretreatment with 5% O_2_ for 48 h on BM-MSC apoptosis by flow cytometry. The results indicated that the percentage of positive (Annexin V^+^, PI^+^) cells after hypoxia pretreatment was similar to those cultured under normoxic conditions (Fig 1D). Moreover, hypoxic pretreatment had no effect on the cell phenotype or adipogenic and osteogenic differentiation abilities. We further examined the phenotypic changes of BM-MSCs under 5% oxygen pretreatment and found that the phenotype of BM-MSCs showed no obvious change after hypoxic preconditioning (S1 Fig). Western blotting indicated that pretreated BM-MSCs exhibited enhanced expression of the anti-apoptotic protein Bcl-2 (1:500, Cell Signaling), while the expression of apoptotic protein caspase-3 (1:500, Cell Signaling) was not altered (Fig 1C).

### Hypoxia pretreatment of BM-MSCs improves the function of HUVECs in vitro

**Fig 2:**
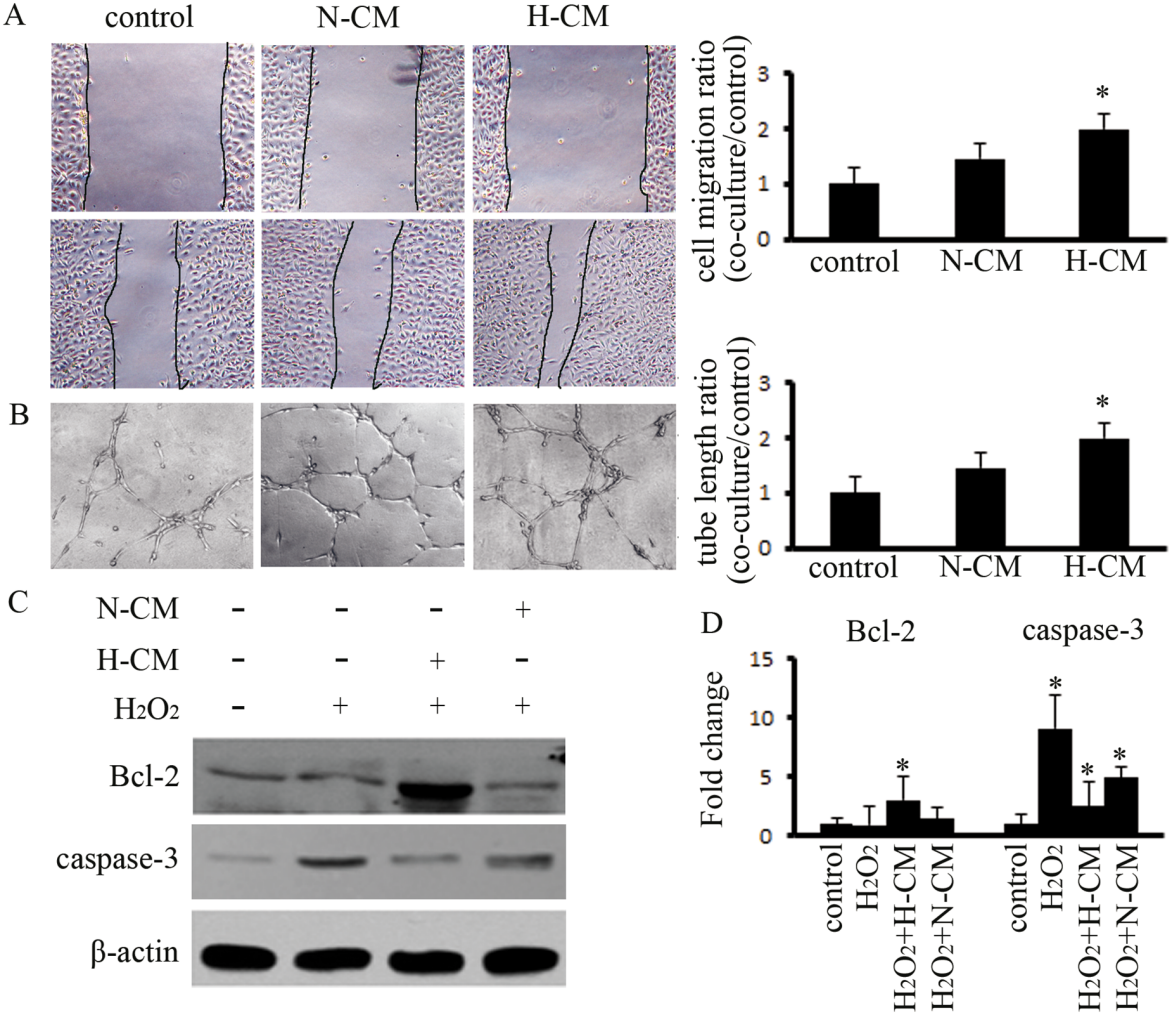
Hypoxia pretreatment of BM-MSCs enhanced the capacity for angiogenesis and reduced apoptosis in HUVECs. (A) Hypoxia pretreatment of BM-MSCs enhanced the capacity for migration by HUVECs after 12 h, as compared to the control group; the efficiency of cell migration was detected. * indicates *P*<0.05 versus control group. (B) Hypoxia pretreatment of BM-MSCs increased the ability for tubular formation by HUVECs after 24 h, as compared to the control group; the average length of the tubular formations was calculated. * indicates *P*<0.05 versus control group. (C,D) H_2_O_2_-treated HUVECs exhibited high expression of caspase-3. The CM from hypoxia-pretreated BM-MSCs depressed the expression of caspase-3 and enhanced the expression of Bcl-2. * indicates *P*<0.05 versus control group.

Furthermore, we observed the effects of hypoxia pretreatment of BM-MSCs on ECs *in vitro*. Conditioned medium (CM) was prepared from hypoxia-pretreated BM-MSCs that were concentrated 10 times and were used to evaluate the paracrine effect of BM-MSCs on HUVECs as well as the function of CM *in vivo* (S2 Fig). The results indicated that non-concentrated CM from normoxic BM-MSCs (N-CM) increased the capacity of migration and tube formation on HUVECs as compared to the control group, and this capacity was significantly increased after treatment with CM from hypoxia-pretreated BM-MSCs (H-CM) (Fig 2A and 2B). Increasing the anti-apoptotic function of ECs is important for angiogenesis. Therefore, we investigated the effects of CM on HUVECs apoptosis. The results indicated that H-CM downregulated the expression of caspase-3, which is induced at 4 h by H_2_O_2_, and upregulated the expression of Bcl-2 (Fig 2C and 2D). Therefore, H-CM improved the function of HUVECs *in vitro*.

### Hypoxia-pretreated BM-MSCs activate VEGF/AKT signaling in HUVECs

**Fig 3:**
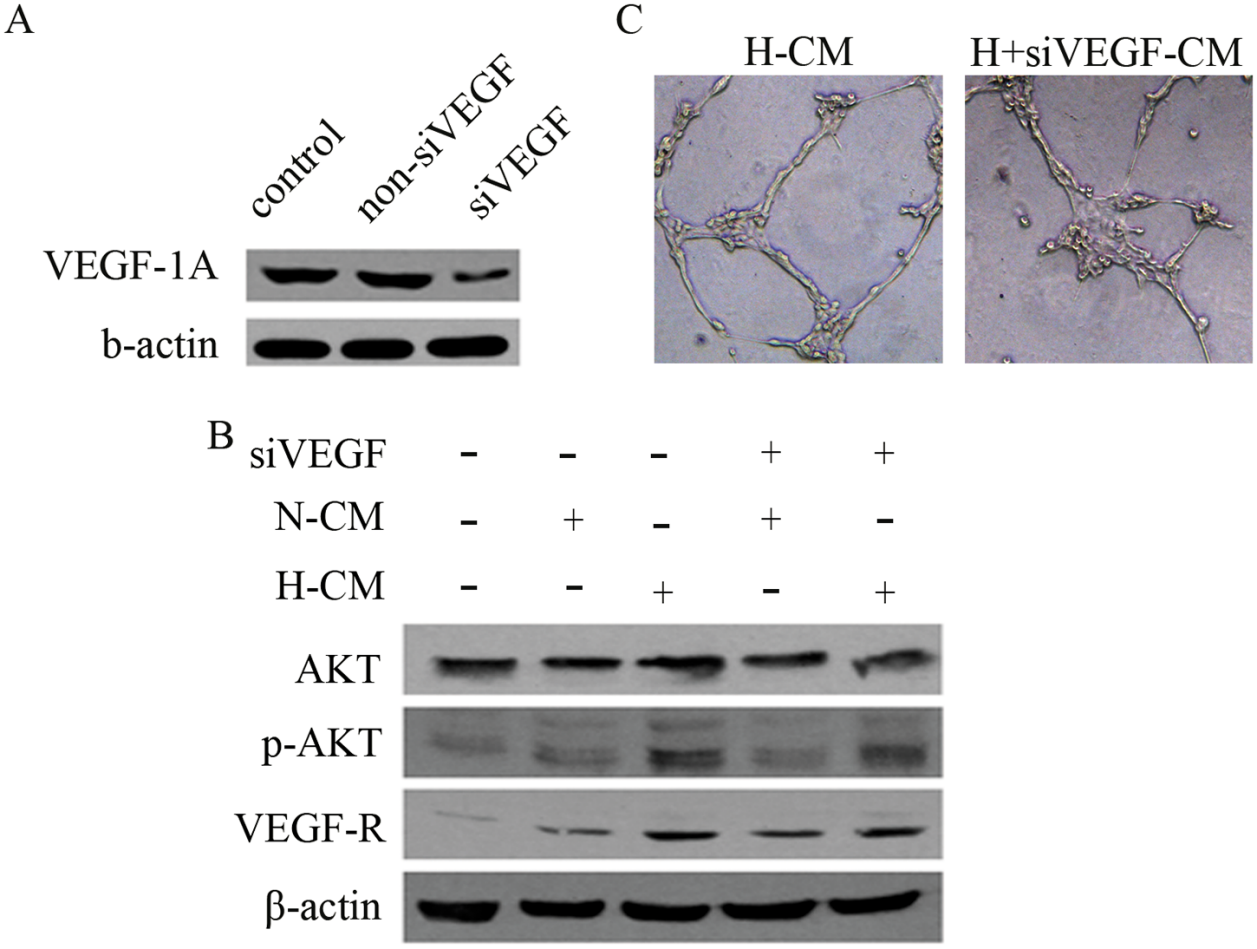
Hypoxia pretreatment of BM-MSCs modulates VEGF/AKT signaling in HUVECs. (A) The expression of VEGF-1α was suppressed with lentiviral-mediated siRNA-VEGF-1α (siVEGF). (B) CM generated from hypoxia pretreated BM-MSCs treated with siVEGF downregulated the expression of pAKT and VEGFR in HUVECs, as determined by western blot. (C) The capacity of HUVECs to form tubes was inhibited by CM from VEGF-1α-silenced BM-MSCs.

The expressions of VEGF/AKT-related proteins in endothelial cells were affected by hypoxia pretreatment of BM-MSCs, as assessed by western blot. The results indicated that the expression of pAKT and VEGF-R in ECs was upregulated with H-CM. However, when the expression of VEGF-1α was silenced in BM-MSCs by lentivirus-mediated siRNA (Fig 3A), the upregulated expression of VEGFR and pAKT was suppressed in HUVECs treated with H-siVEGF-CM. (Fig 3B). Furthermore, the capacity for tube formation by HUVECs was inhibited by H-siVEGF-CM treatment (Fig 3C). The expression of VEGF/AKT-related protein in ECs was activated by BM-MSCs, and the capacity for angiogenesis was improved.

### Hypoxia pretreatment of BM-MSCs promotes angiogenesis in DLLI rats

**Fig 4:**
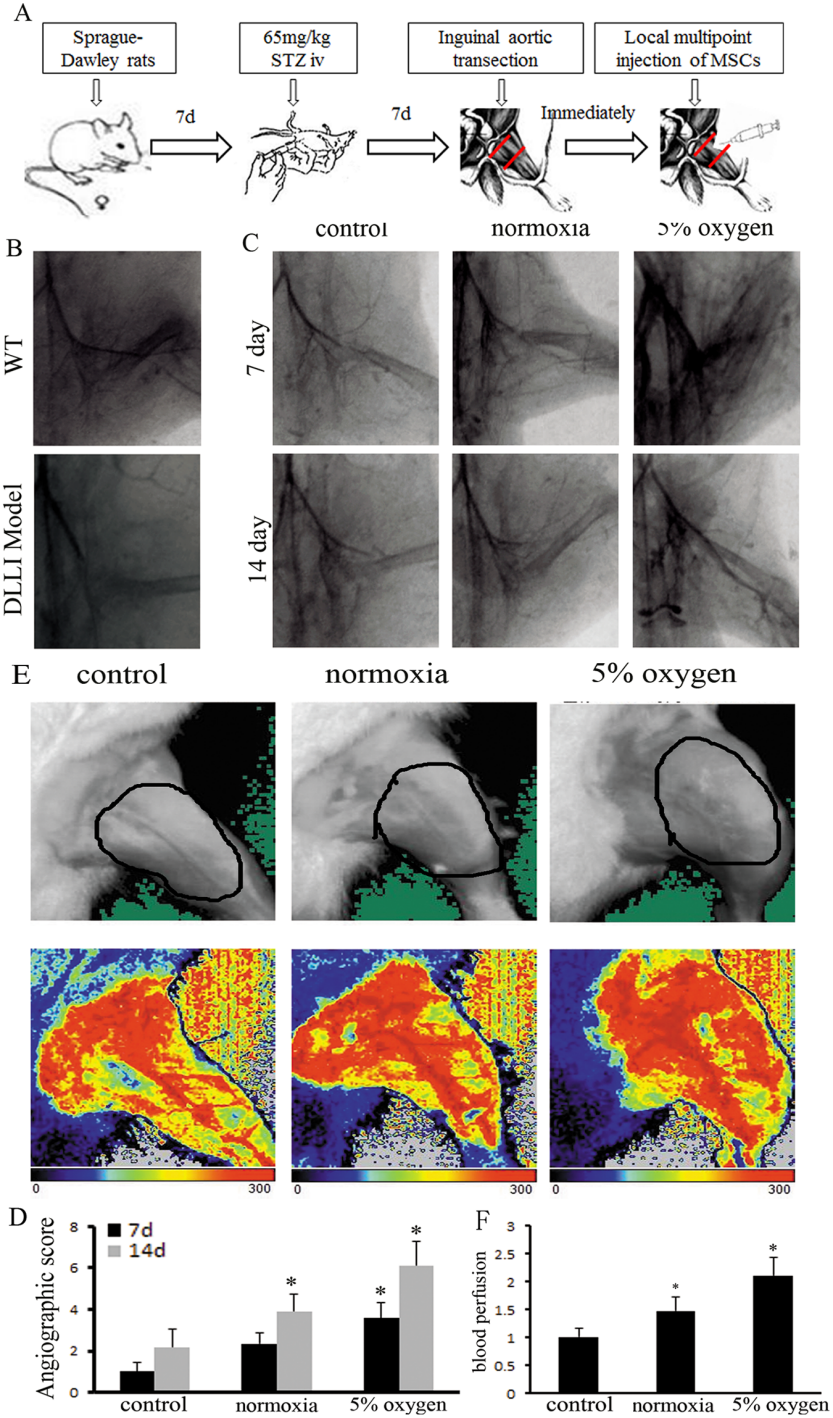
Hypoxia pretreatment of BM-MSCs promoted angiogenesis in ischemic muscle, as evaluated by angiography. (A) We presented the block diagram of study for BM-MSCs transplanted to treat DLLI. (B) The model of DLLI was developed and confirmed by angiography. (C) Hypoxia pretreatment of BM-MSCs locally transplanted to ischemic muscle markedly enhanced the microvascular density, compared to the control group. (D) The average length of the capillaries was detected. *n* = 4. * indicates *P*<0.05 versus the 7 d control group. (E) Perfusion images of lower limb at 14 days postoperatively. The color scale of all images was analyzed by setting the lowest perfusion value to 0 and the highest perfusion value to 300. (F)Perfusion in the normoxia and control groups was lower than that in the hypoxia group (* indicates *P<0*.*05*).

The DLLI model, established by STZ-induced diabetic rats with ligation of the femoral artery, was confirmed by angiography (Fig 4A). Hypoxia-pretreated BM-MSCs were injected into ischemic muscle tissue along the artery with multiple local injection points, and normoxic BM-MSCs and saline were used in the control groups. Seven days after cell transplantation, angiography indicated that neovascularization was found at ischemic tissue in the hypoxia pretreatment group, and that the mean length of the capillary was greater than that of the control groups. At day 14, a renascent vascular network had been formed at the femoral artery break away site in the hypoxia-pretreated group (Fig 4B–4D). Laser Doppler perfusion imaging was performed 14 days postoperatively to detect the blood perfusion of the ischemia of lower extremities. Compared with the hypoxia group, perfusion was decreased in the normoxia and control groups (Fig 4E and 4F). In the process of cell therapy, body weight, blood glucose, and glycosylated hemoglobin (HbA1c) levels were measured, and the results showed insignificant differences in local transplantation in the BM-MSCs and control groups (Table 2).

**Table 2:** The biochemical index of the rats.

	Bodyweight(g)			bloodglucose(mmol/L)			HbA1c(%)		
**group**	3day	7day	14day	3day	7day	14day	3day	7day	14day
**control**	251±3	228±2	180±5	28±2	30±3	29±3	11.8±0.3	12.1±0.2	12.2±0.3
**normoxia**	251±5	225±4	182±3	27±2	27±3	30±1	12.1±0.3	11.9±0.2	11.9±0.4
**hypoxia**	251±4	232±4	175±7	30±5	29±4	29±1	12.0±0.4	12.0±0.2	12.0±0.1

**Fig 5:**
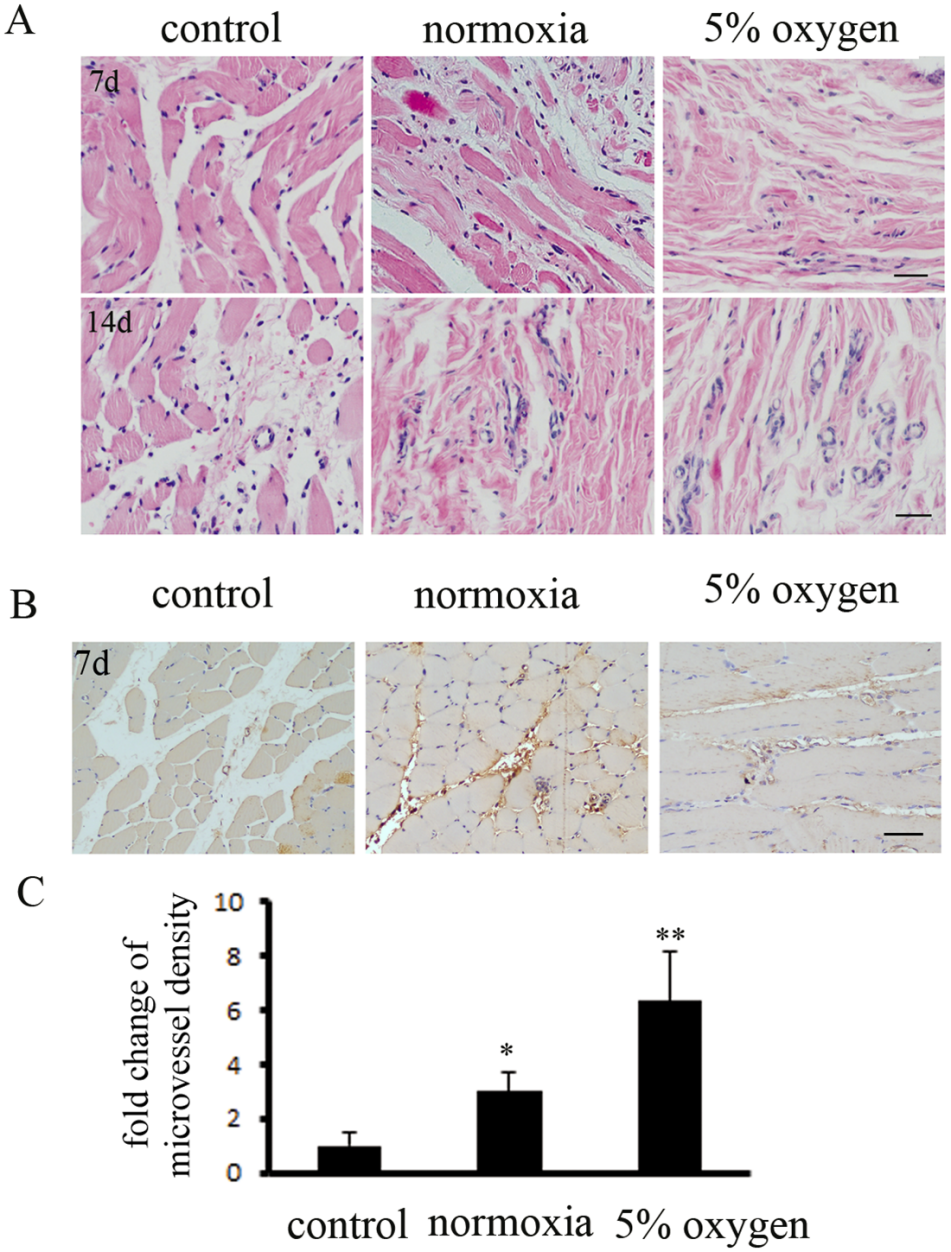
Hypoxia pretreatment of BM-MSCs promoted angiogenesis in DLLI muscle, as assessed by immunohistochemical staining. (A) Histological analysis of muscle tissues was performed by immunostaining. The specimens were stained with H&E (hematoxylin-eosin). Hypoxia pretreatment of BM-MSCs accelerated the repair of muscle fibers at day 7. At day 14, the hypoxia pretreatment BM-MSCs group exhibited a de novo rebound in the microvasculature. (B) Immunohistochemical staining with a specific antibody recognizing the endothelial marker CD31 revealed the presence of more mature vessels (muscle), predominantly in the hypoxia pretreatment BM-MSCs group rather than in the control group, and (C) the number of vessels was measured.*, ** indicate *P*<0.05, 0.01 versus control group, respectively.

The ischemic muscle tissues were collected for paraffin sections. H&E staining indicated that hypoxia pretreatment of BM-MSCs significantly repaired muscle fibers in ischemic tissue at 7 d and 14 d, compared to the other groups (Fig 5A). Next, immunohistochemical staining demonstrated that the mature microvessel density, counted by immunostaining of endothelial cells marked with anti- CD31 antibodyies, was markedly increased in the hypoxia pretreatment BM-MSCs group at 7 d (Fig 5B and 5C). Hypoxia pretreatment of BM-MSCs increased the repair of ischemic tissues in DLLI.

### Hypoxia pretreatment enhances the survival of BM-MSCs and promotes the expression of angiogenic factors in ischemic tissue

**Fig 6:**
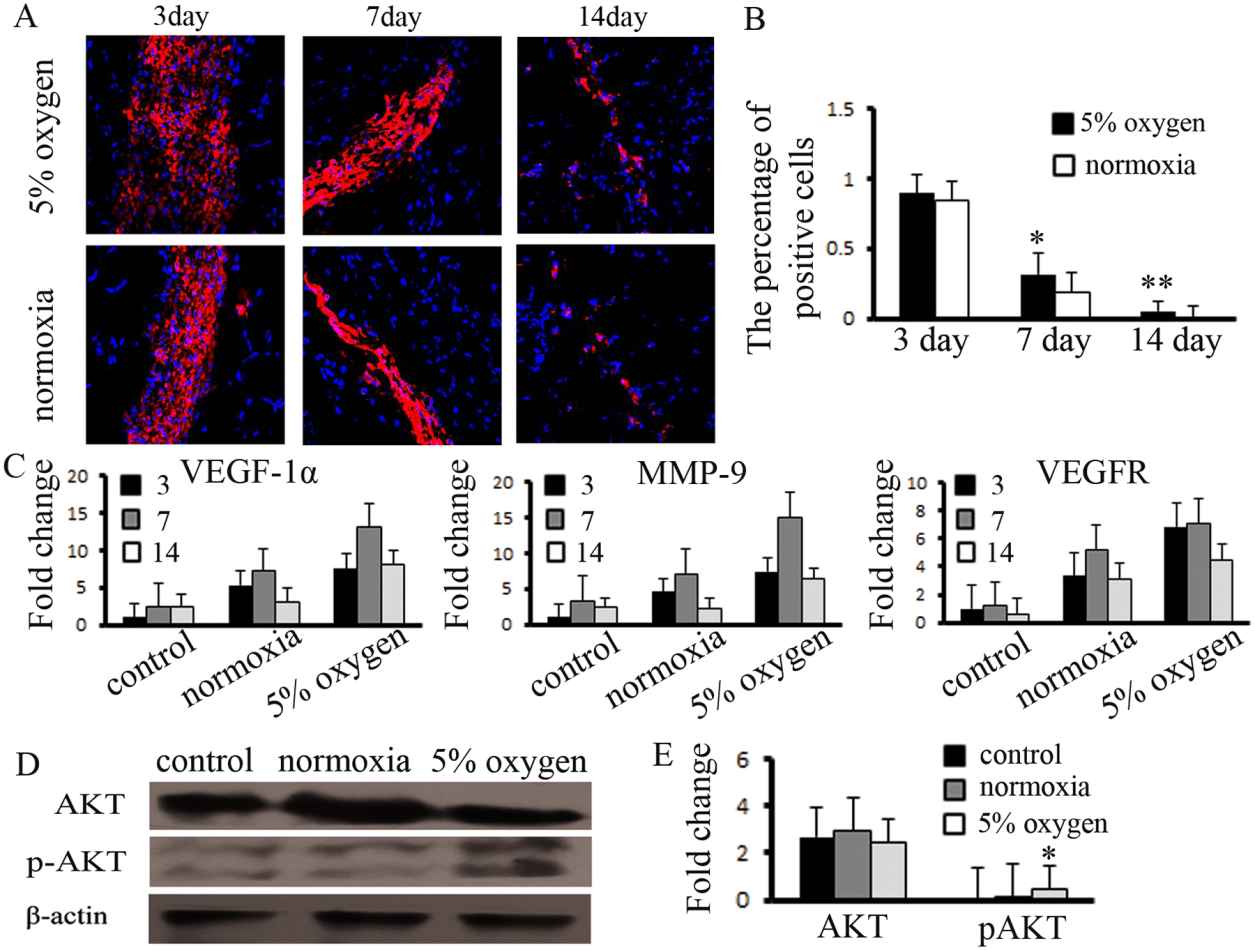
Hypoxia pretreatment promoted the function of BM-MSCs in ischemic muscle. (A) Hypoxia pretreatment of BM-MSCs increased the capacity for survival in ischemic muscle, compared to the normoxia group, (B) and the number of positive cells was measured. *, ** indicate *P*<0.05, 0.01 versus normoxia group, respectively. (C) The expression of VEGF-1α, MMP-9, and VEGFR was increased in hypoxia-pretreated BM-MSCs, and (D, E) the expression of pAKT was significantly enhanced, compared to the control group. * indicates *P*<0.05 versus control group.

We confirmed that hypoxia pretreatment of BM-MSCs activated the expression of anti-apoptotic proteins *in vitro*. Based on this result, we analyzed the survival of BM-MSCs *in vivo*. The cell tracer results indicated that there were a similar number of cells in the three groups at 3 d. Thereafter, the survival of the hypoxia-pretreated BM-MSCs was higher than that of the normoxia group. Until day 14, approximately 5% of the hypoxia-pretreated BM-MSCs remained, whereas no surviving cells were observed in the normoxia group (Fig 6A and 6B). We further detected the survival of BM-MSCs in non-ischemic muscle, the results shows that BM-MSCs reduced survival ability compared with the ischemia group (S3 Fig). We also detected the expression of antigenic factors in the ischemic tissues. The results indicated that the expression of angiogenic factors was significantly increased in the hypoxia-pretreated BM-MSC group at days 3, 7, and 14 (Fig 6C). However, while the survival of BM-MSCs was enhanced by hypoxia pretreatment in vivo, we examined the expression of downstream target proteins mediated by proangiogenesis factors and found that the expression of pAKT (1:400, Cell Signaling) was upregulated and that the expression of AKT (1:600, Cell Signaling) exhibited no changes at day 7 (Fig 6D and 6E). Hypoxia pretreatment of BM-MSCs promoted the survival ability of the injected cells, increased the expression of angiogenic factors in ischemic tissue and activated the expression of angiogenesis-related signals.

### Hypoxia pretreatment of BM-MSCs improves endothelial function in ischemic tissues

**Fig 7:**
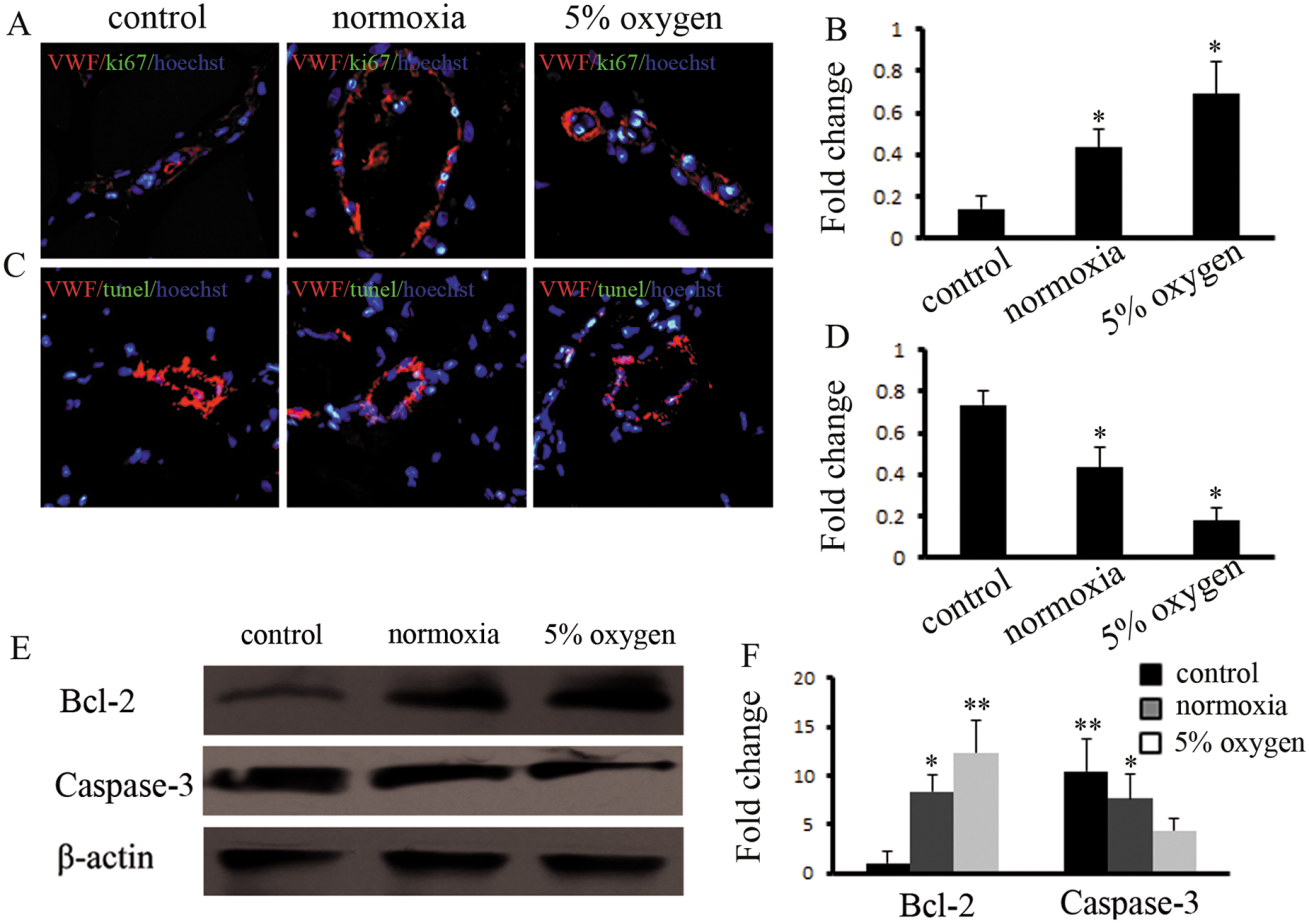
Hypoxia pretreatment of BM-MSCs improved the function of ECs in vivo. (A, B) Immunohistochemical staining with specific antibodies against the endothelial marker vWF and cell proliferation marker Ki67 revealed more double-positive cells in the hypoxia pretreated BM-MSC group than in the control group. * indicates *P*<0.05 versus control group. (C, D) Immunohistochemical staining with a specific antibody recognizing the endothelial marker vWF and cell apoptosis marker TUNEL revealed that hypoxia pretreatment of BM-MSCs decreased the number of double-positive cells. * indicates *P*<0.05 versus control group. (E,F) Hypoxia pretreatment of BM-MSCs upregulated the expression of Bcl-2 in muscle and reduced the expression of caspase-3. * indicates *P*<0.05 versus control group. * indicates *P*<0.05 versus control group.

We further analyzed the function of endothelial cells with BM-MSCs in ischemic tissue by the hypoxia pretreatment of BM-MSCs. Greater numbers of ECs triple-stained for proliferation and apoptosis were found in the ischemic tissues of DLLI rats administered normoxia-or hypoxia-pretreated BM-MSCs, compared with the saline group (Fig 7A and 7B). The results indicated that the number of positive cells triple-stained with vWF/Ki67/Hoechst 33342 were significantly greater in the hypoxia-pretreated BM-MSC group than in the normoxia and saline groups. In contrast, the number of vWF/TUNEL/Hoechst 33342 triple-stained positive cells decreased in the hypoxia pretreatment group compared with the normoxia and saline groups (Fig 7C and 7D). The expression of apoptotic and anti-apoptotic proteins was also detected in the ischemic tissue of DLLI rats by western blotting. The results suggested that the expression of Caspase-3 was downregulated in the hypoxia-pretreated BM-MSC group, while the expression of Bcl-2 was upregulated (Fig 7E and 7F). The results demonstrated that hypoxic pretreatment of BM-MSCs promoted ECs proliferation and decreased apoptosis of ECs.

## Discussion

**Fig 8:**
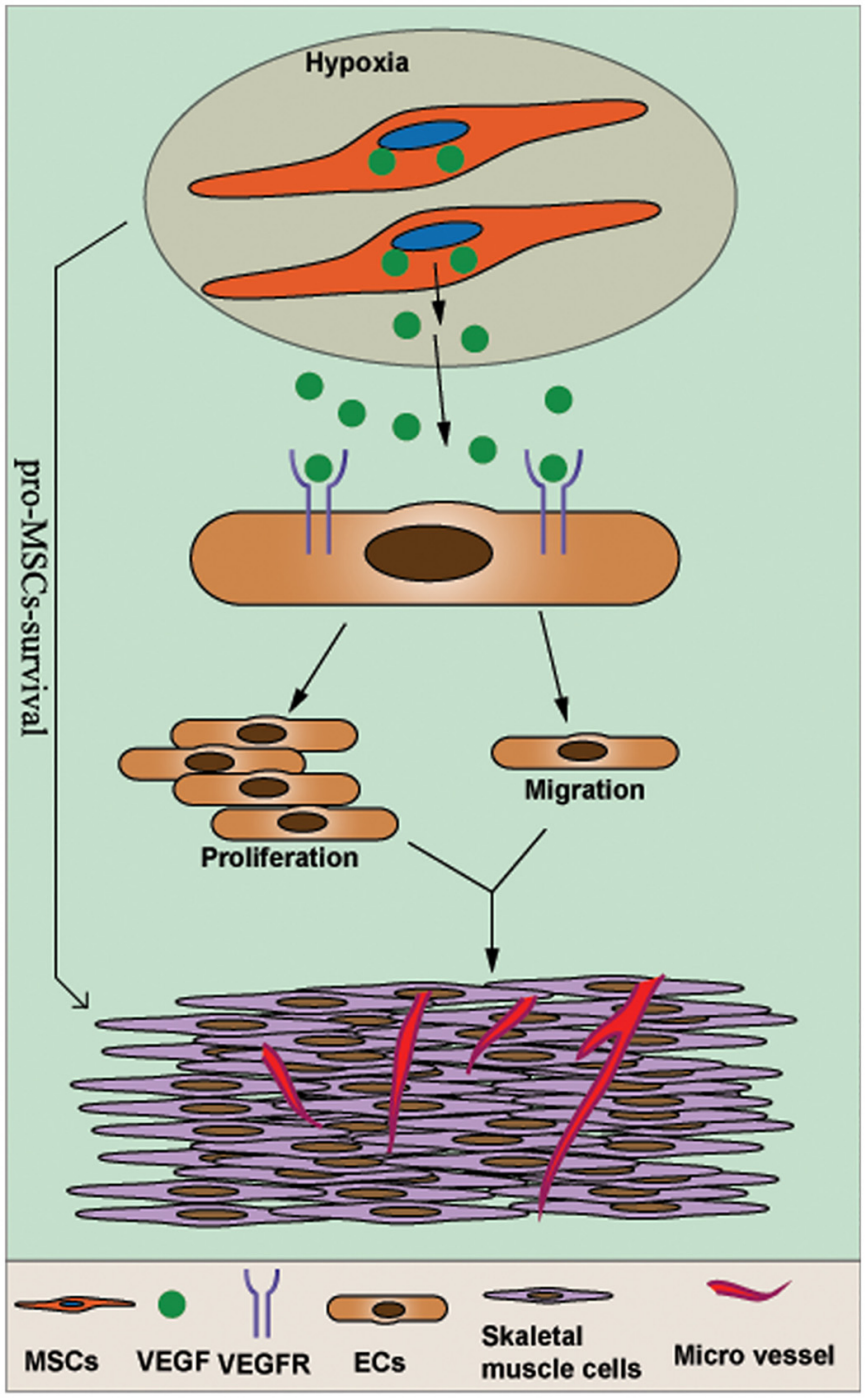
Hypoxia-pretreated BM-MSCs significantly increased the survival of the BM-MSCs in ischemic muscle, markedly increased angiogenesis and improved the proliferation of endothelial cells in ischemic muscle.

In this study, we confirmed that BM-MSCs cultured with 5% O_2_ for 48 h exhibited significantly improved capacity for the secretion of angiogenic factors. Local transplantation of hypoxia-pretreated BM-MSCs significantly increased the survival of the BM-MSCs in ischemic muscle, markedly increased angiogenesis and improved the proliferation of endothelial cells in ischemic muscle (Fig 8) *In vitro*, we also confirmed that the hypoxia pretreatment of BM-MSCs increased angiogenesis by ECs.

Currently, endothelial dysfunction is a systemic pathological condition that can be broadly defined as an imbalance between vasodilating and vasoconstricting substances manufactured by the endothelium or inclusive functions of the endothelium [27]. Diabetic mellitus generates the disordered metabolism of proteins and fats due to inappropriate production of insulin, a blood glucose regulator, or resistance to insulin that results in high blood glucose levels, or hyperglycemia [28]. Over time, elevated glucose levels in the bloodstream can lead to the impaired function of the vessel endothelial cells [29]. Nevertheless, owing to endothelial dysfunction, the *de novo* endothelial cells cannot expand, leading to difficulties in neovascularization or angiogenesis that are also interchangeably associated with vasculogenesis, which primarily refers to the developmental formation of vascular structures. A previous study confirmed that MSCs ameliorate DLLI by promoting ischemic tissue angiogenesis by improving the function of endothelial cells [30, 31]. In our study, we confirmed that the generated MSCs marked the improvement of angiogenesis in DLLI by promoting the proliferation and inhibiting the apoptosis of endothelial cells. Furthermore, the hypoxia pretreatment of MSCs was able to significantly enhance restorative effects.

Mesenchymal stem cells have been isolated from bone marrow, adipose tissue, and other adult tissues [32]. However, the number of MSCs contained in tissues is low, and the cells must be expanded *in vitro* for transplantation. In this process, BM-MSCs usually encounter two distinct environmental conditions. One is the *in vitro* culture environment (from isolation to transplantation) and the other is the physiological conditions *in vivo* (before isolation and after transplantation). At present, most of the expansion procedures for BM-MSCs are performed under ambient O_2_ concentration, in which cells are exposed to 21% O_2_ [33]. It is well known that MSCs exist in hypoxic physiologic conditions, and that the cells are quiescent. The cells do not proliferate and maintain a potential pluripotent ability in this period. Several studies have presented positive evidences regarding the adverse effects of ambient O_2_ concentration on BM-MSCs, including senescence, population doubling time, DNA damage, and poor engagement following transplantation [22, 24]. However, murine MSCs preconditioned in a hypoxic environment demonstrated increased skeletal muscle regeneration after 7 days, with improved blood flow and vascular formation compared to MSCs maintained in normoxic conditions [34]. Hypoxia plays a critical role in maintaining homeostasis within the body from the very beginning of embryonic development and helps to facilitate proper embryonic progress, maintain stem cell pluripotency and regulate the signaling of multiple cascades, including angiogenesis. All of these effects reflect the influential effect of the O_2_ concentration on BM-MSC biology and raise serious concerns over the impact of the O_2_ concentration on therapeutic efficiency, biosafety, cell proliferation and maintenance of homeostasis. In this study, we proved that BM-MSCs cultured with 5% O_2_ for 48 h were able to dramatically enhance the anti-apoptotic activity of endothelial cells, while having no effects on cell proliferation and apoptosis, and were also able to increase the competence of secreted proangiogenic factors.

MSCs secrete angiogenic cytokines, such as VEGF and HGF, which may contribute to their angiogenic properties. A number of studies have shown that MSCs secrete significant quantities of angiogenic and anti-apoptotic factors, including VEGF and HGF [24]. These findings further encouraged a series of *in vivo* studies that focused on evaluating the therapeutic potential of cells based particularly on their paracrine and angiogenic effects. The processes of neo-vascularization and angiogenesis, including VEGF and bFGF, are regulated by a number of endothelial growth factors. Furthermore, pathological conditions, such as ischemia, induce the release of bFGF and VEGF from MSCs, leading to the formation of new vascular vessels [35]. VEGF is an important angiogenic factor and promotes the proliferation and migration of ECs maintaining the integrity of vascular vessels. VEGF facilitates angiogenesis synergistically with angiogenin was proved in the past investigation. The growth factor affects early blood vessel formation and promotes the generation of a primitive vascular network, while angiopoietin acts on the subsequent alterations of vascular remodeling and promotes the formation of mature vessels and the spatial structure of the vascular network. In a recent study, conditioned medium from hypoxic MSC prevented endothelial cell apoptosis and enhanced tube formation *in vitro* [36]. In addition, when ischemic muscles were treated with conditioned medium harvested from MSCs cultured under hypoxic conditions, the experimental animals recovered faster than the control groups, suggesting that the release of angiogenic factors by MSCs was sufficient to enhance the revascularization of the injured tissue. In all of these studies, however, the conditioned media or MSCs were injected directly into the injured muscle, which eliminated the necessity of MSCs to home to the site of injury.

We measured the percentage of MSCs remaining at the site of injury 2 weeks after injection. The function of the cells is to release cascades of trophic factors to enhance the endogenous revascularization process rather than the recreation of new vessels or muscle. Therefore, from a clinical and regulatory agency point of view, it is actually preferable that the cells do not remain in the local area for months after the injury has been repaired. This finding could enhance therapeutic approaches for enhancing local tissue repair by injected human mesenchymal stem cells.

## Conclusions

In our study, we demonstrated that hypoxia pretreatment of MSCs significantly enhances the secretion of bioactive factors, extending the efficiency of cell survival after transplantation into ischemic muscle in DLLI. Our findings show that MSC pretreatment may be a novel strategy for the clinical treatment of diabetic lower extremity arterial disease.

## Supporting Information

S1 Fig The phenotype and differentiation of BM-MSCs cultured under hypoxic conditions. (A) BM-MSCs surface marker expression by flow cytometry showing the percentages of BM-MSCs cultured with 5% O_2_ 48 h for mesenchymal antigens has similar to the normoxia group. (B) Multilineage differentiation potential of BM-MSCs showing BM-MSCs differentiated into adipocytes, which are indicated by the accumulation of lipid vesicles in the cells, (C) and osteoblasts, which express alkaline phosphatase, as indicated in blue. Scale bar = 100 μm.
(TIF)

S2 Fig The CM from the hypoxia pretreated of BM-MSCs improved angiogenesis in vivo. (A) The result of angiography showed that the CM from hypoxia pretreated of MSCs improved angiogenesis in DLLI at day 14, (B) and the number of positive cells was measured. * indicate P<0.05, 0.01 versus normoxia group.
(TIF)

S3 Fig The MSCs transplanted to non-ischemic lower limb in diabetic rats. (A) The result of the CM-Dil label MSCs by fluorescence microscope.
(TIF)
