# Supplementary figures and images for: Hypoxia Pretreatment of Bone Marrow Mesenchymal Stem Cells Facilitates Angiogenesis by Improving the Function of Endothelial Cells in Diabetic Rats with Lower Ischemia
Source: PLoS One. 2015 May 21;10(5):e0126715. doi: 10.1371/journal.pone.0126715 (PMC4440823; doi:10.1371/journal.pone.0126715)

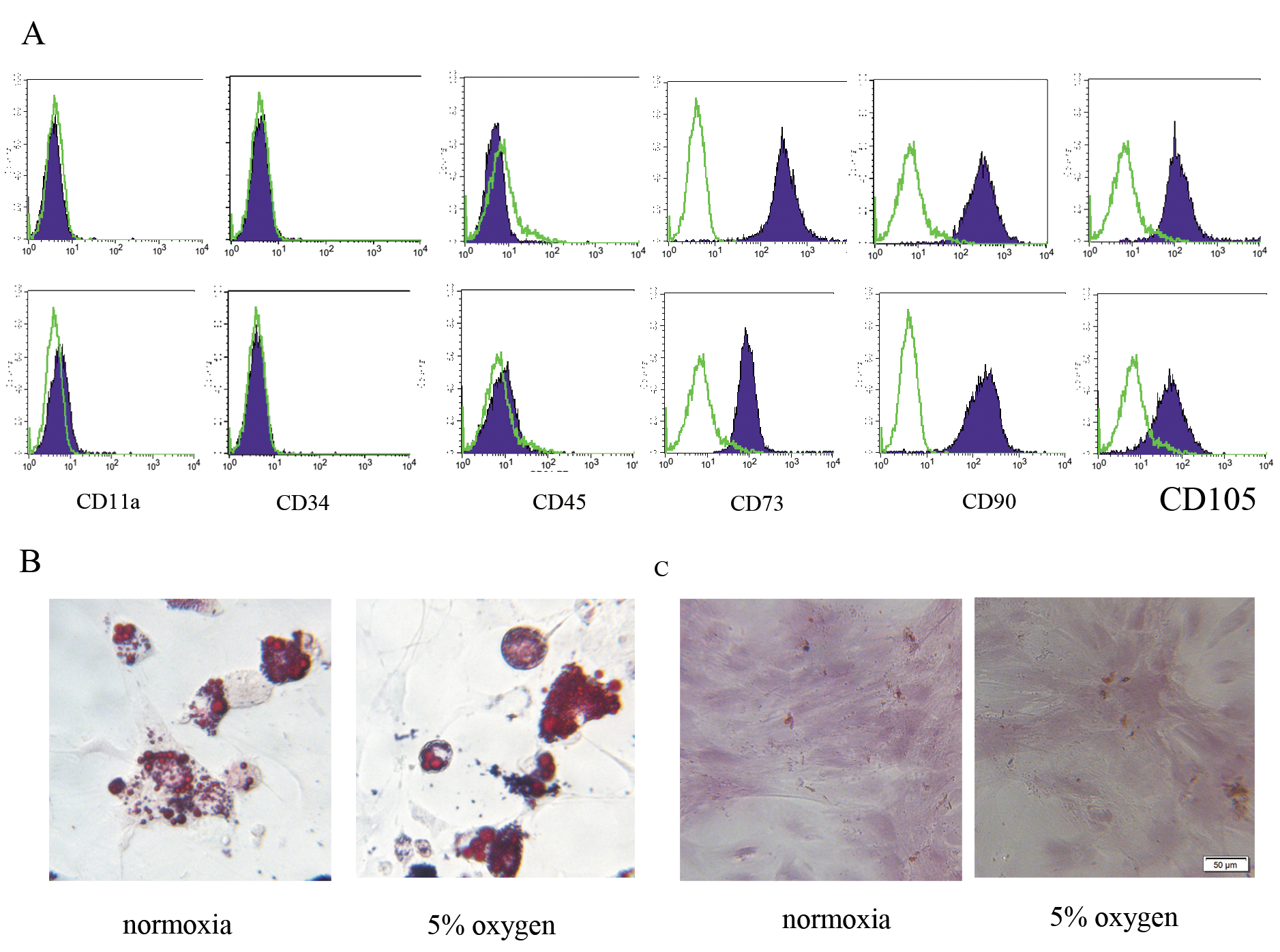

Supplement: S1 Fig — (A) BM-MSCs surface marker expression by flow cytometry showing the percentages of BM-MSCs cultured with 5% O2 48 h for mesenchymal antigens has similar to the normoxia group. (B) Multilineage differentiation potential of BM-MSCs showing BM-MSCs differentiated into adipocytes, which are indicated by the accumulation of lipid vesicles in the cells, (C) and osteoblasts, which express alkaline phosphatase, as indicated in blue. Scale bar = 100 μm. (TIF) [file pone.0126715.s001.tif]

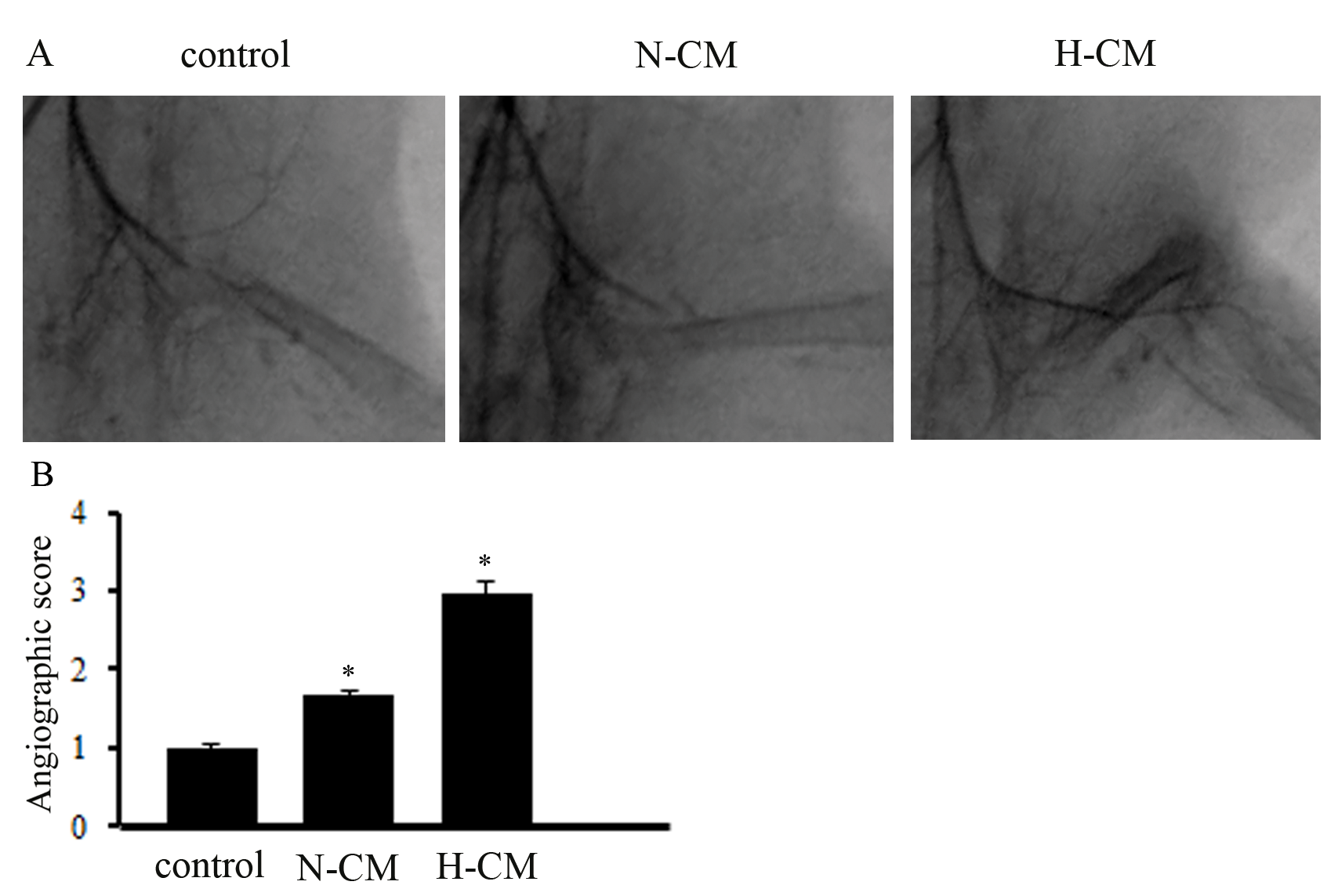

Supplement: S2 Fig — (A) The result of angiography showed that the CM from hypoxia pretreated of MSCs improved angiogenesis in DLLI at day 14, (B) and the number of positive cells was measured. * indicate P<0.05, 0.01 versus normoxia group. (TIF) [file pone.0126715.s002.tif]

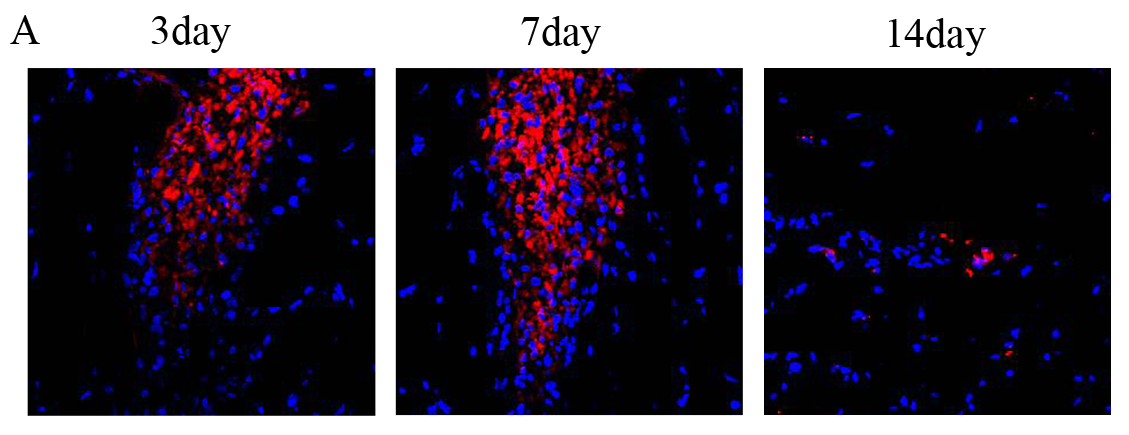

Supplement: S3 Fig — (A) The result of the CM-Dil label MSCs by fluorescence microscope. (TIF) [file pone.0126715.s003.tif]
